# Isotopic evidence for human mobility in late antique Bulla Regia (Tunisia)

**DOI:** 10.1016/j.jasrep.2022.103816

**Published:** 2023-02

**Authors:** Efthymia Nikita, Michelle Alexander, Samantha Cox, Anita Radini, Petrus Le Roux, Moheddine Chaouali, Corisande Fenwick

**Affiliations:** a Science and Technology in Archaeology and Culture Research Centre, The Cyprus Institute, 2121 Nicosia, Cyprus; b BioArCh, Department of Archaeology, University of York, YO1 5DD York, UK; c Department of Genetics, Perelman School of Medicine, University of Pennsylvania, Philadelphia, PA 19104, USA; d Physical Anthropology Section, Penn Museum, University of Pennsylvania, Philadelphia, PA 19104, USA; e Department of Geological Sciences, University of Cape Town, Rondebosch 7701, South Africa; f Institut National du Patrimoine, 1008 Tunis, Tunisia; g Institute of Archaeology, University College London, WC1H 0PY London, UK

**Keywords:** Palaeomobility, Tunisia, Roman, Late antiquity, Isotopes

## Abstract

This paper represents the first isotopic study on late antique human mobility in North Africa, using the urban site of Bulla Regia in Tunisia as a case study. We also present the first values for bioavailable ^87^Sr/^86^Sr in northern Tunisia, analysing 63 plant and snail samples, as well as a simple method for the pre-processing of plants in the field to facilitate their export. Bulla Regia was a prominent Roman and late antique town situated on an important axis of transport and communication in North Africa and is therefore an ideal site to explore mobility in the region during this time period. Strontium (^87^Sr/^86^Sr) and oxygen (δ^18^O_Carb_) isotopic analysis of 22 late antique individuals from a Christian church and cemetery identified at least seven or eight non-locals, while comparative analysis of five Roman individuals from a funerary enclosure on the same site classified all but one of them as potential locals. Most non-local individuals exhibit ^87^Sr/^86^Sr values that match various areas of northern Tunisia, which supports regional mobility rather than long-distance migration, although when combined with the oxygen results, inter-regional mobility from an area with a warmer climate may be hypothesised for some individuals. Examination of the spatial distribution of non-local individuals in their cemetery setting reveals that they were privileged individuals, thus they may reflect the mobility of wealthier town-dwellers in late antiquity, particularly perhaps along the Carthage-Hippo route.

## Highlights

• First isotopic study on North African late antique mobility; Bulla Regia case study.
• 7 out of 22 late antique individuals nonlocal; 4 out of 5 comparative Romans local.
• Nonlocals could have originated in northern Tunisia, hence regional mobility.
• Inter-regional mobility from warmer areas possible for some individuals.
• First published values for bioavailable ^87^Sr/^86^Sr in northern Tunisia.

## 1 Introduction

Tunisia has been a focal point of interaction for people from Africa, Europe, and the Middle East since prehistory. Modern genetic studies support significant heterogeneity in the structure of the population, and a varying impact of admixture and isolation (Anagnostou et al., 2020, Cherni et al., 2009, Cherni et al., 2016; but see also Fadhlaoui-Zid et al., 2011).

Late antique North Africa was characterised by successive conquests by the Vandals, Byzantines and Arabs, and large-scale inward movement of soldiers, administrators and merchants, occasionally accompanied by their families, some of whom settled in the region. The Vandals joined by Alans first arrived in Africa in 429 CE, captured Carthage in 439 and by 442, ruled Africa Proconsularis, Byzacena, Tripolitania, and Numidia through a treaty with the imperial government. In 533–4 North Africa was captured by the armies of Justinian I and became part of the Roman Empire again, though the number of newcomers settling permanently was probably limited (Conant, 2012). Umayyad armies first entered Africa in 642, Carthage fell in 697/8 and the region became Ifriqiya and part of the Umayyad and then Abbasid Caliphates, before becoming an independent vassal state under the Aghlabid dynasty in 800 (Fenwick, 2020). Throughout these political changes, North Africa continued to be one of the most prosperous Mediterranean regions (e.g. Hobson, 2015, Leone, 2007, Sears, 2007) which exported its grain, olive oil, ceramics and other goods across the Mediterranean into the seventh century and beyond. As well as the large-scale mobility of soldiers, settlers and merchants instigated by the Vandal, Byzantine and Arab conquests, trade and maritime activity underpinned a constant level of regional mobility along the African coastline and intra-regional mobility with the Mediterranean, particularly with Sicily, Italy and Iberia (McCormick, 2001). Merrills (2018) has also highlighted the importance that seasonal regional and intra-regional mobility played in the political and social upheavals of late antiquity. Such seasonal mobility was necessitated by the rhythm of the agricultural year, where the harvest period and its manpower demands would attract seasonal pastoralists as well as groups of workers who moved from towns to the countryside, and between estates (Shaw, 2013, Tedesco, 2018). Finally, historical sources attest to the continued frequent regional movement of individuals in North Africa for other reasons, e.g. priests and bishops such as St Augustine (Shaw, 2011).

This paper explores patterns of human mobility in late antique Tunisia, employing strontium (^87^Sr/^86^Sr) and oxygen (δ^18^O_Carb_) isotope analysis to analyse a skeletal assemblage from Bulla Regia. This site offers an ideal opportunity to explore whether hypothesised elevated human mobility is reflected in the burial population. Bulla Regia is situated in the Central Medjerda Valley, a key grain-producing region in the Roman and medieval periods and an important axis of transport and communication between Carthage/Tunis, Hippo Regius (Annaba) and Cirta/Constantine further west. The site was established no later than the 4th century BCE and is thought to have been one of the residences of the Numidian rulers. Following the Roman conquest of North Africa, the town became an *oppidum liberum*, and was quickly elevated to *municipium* under Vespasian, and to *colonia* under Hadrian. In the early Roman period, there was a good degree of mobility as shown by the number of senators and equestrians the town provided to Rome during this period (Thébert, 1973). Yvon Thébert’s onomastic study of Bulla Regia’s funerary and dedicatory inscriptions suggests that the Roman population was largely indigenous, and provides limited evidence of immigrants. However, he also notes the relatively common qualification of local notables as *alumnus*, which signifies that an individual has been brought up since infancy at Bulla Regia, but not born there. Reasons for this would have varied but may have included infants born on estates or while their father was serving appointment elsewhere (Thébert, 1973). Our understanding of late antique Bulla Regia is less certain, though it remained a wealthy and well-connected town, as attested by the presence of several bishops who attended the conferences in Carthage (Fenwick et al., 2022).

Bioarchaeological methods offer great potential for mapping and understanding the scale of mobility in different periods. Past human mobility can be explored bioarchaeologically using isotopic analyses, ancient DNA (Antonio et al., 2019, Stoneking and Krause, 2011), as well as biodistances (Nikita, 2020, Stojanowski, 2018). However, as yet, bioarchaeological methods for investigating mobility have rarely been used in Tunisia. Only one study has employed ancient DNA to explore mobility. Ancient genomes from the Punic site of Kerkouane in Tunisia (650–250 BCE), indicated mixed ancestry from North Africa, Europe, and the Levant. A number of individuals were generally more similar to modern southern European populations than Tunisians, highlighting the high rates of mobility and admixture along the Tunisian coast prior to the Roman conquest (Moots et al., 2022). Skeletons from Carthage dating from 751 BCE to 435 CE have been used in North African biodistance studies in conjunction with skeletal assemblages from Libya, Sudan, Egypt and Algeria, employing nonmetric and metric data to assess gene flow. The results of these studies showed that the Tunisian individuals clustered with groups from Egypt (Alexandria), Algeria but also with a Sudanese group from Soleb, perhaps thanks to the Mediterranean coast and Nile River that facilitated connectivity (Nikita et al., 2012a, Nikita et al., 2012b). No previous studies have used isotopic analysis to explore mobility patterns, however, the promise of such an approach for late antiquity is demonstrated by recent isotopic analyses of Eastern Mediterranean and southern European assemblages (e.g. Al-Shorman and El-Khouri, 2011, Antonio et al., 2019, de-la-Rua, C., Izagirre, N., Alonso, S., Hervella, M., , 2015, Marcus et al., 2020, Maxwell, 2019, Nikita et al., 2021, Paladin et al., 2020, Perry et al., 2009, Perry et al., 2017, Temkina, 2021, Wong et al., 2018, Zalloua et al., 2018).

Given the major socio-political developments that characterised late antique North Africa, one would anticipate elevated mobility, especially in an important urban centre like Bulla Regia. The present study hence explores a key topic for which there is no prior bioarchaeological information. In the context of this study, we not only elucidate patterns of mobility, identifying potential non-locals and how they map onto the mortuary record, but we also generate important data for future studies in the region.

## 2 Materials and methods

### 2.1 The site of Bulla Regia

Bulla Regia was a wealthy town, renowned for its 4th–5th c. CE underground houses with floor mosaics (Fenwick et al., 2022, Thébert, 1973). To study the development and transformation of Bulla Regia during the transition from Late Antiquity to Middle Ages, the Tunisian–British Bulla Regia Project, a collaboration between the Institut National du Patrimoine (INP) and University College London (UCL), was established in 2014.

**Fig. 1:**
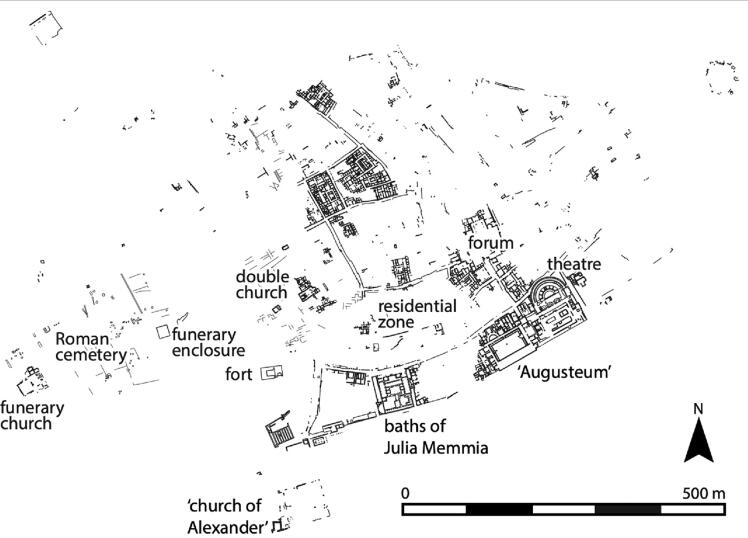
Plan of Bulla Regia showing the location of the late antique church and cemetery and the Roman funerary enclosure (Bulla Regia Archaeological Project).

The material examined in this paper comes from an area known as the western cemetery which contains several visible funerary monuments including early Roman mausolea and a Muslim cemetery and marabout (Chaouali et al., 2018). The western cemetery was first explored in the 1890s by archaeologist Louis Carton, who excavated multiple mausolea as well as simple graves marked by masonry and stone cupulae, steles or cippi, and sometimes placed in walled enclosures (*areae*). Carton discovered some inhumations, however most burials had been cremated *in situ*. The cemetery appears to have been used from the 1st century CE (perhaps earlier) into at least the first quarter of the 4th century (Carton, 1890, 183). During the 1960 s and 1970 s, a French–Tunisian team excavated 10 additional cupola tombs covering cremation burials, which dated to the early 2nd century CE (Khanoussi, 1983). In 2010, a rescue excavation directed by Moheddine Chaouali uncovered a hitherto unknown Roman walled funerary enclosure (1st–4th century) and a late antique Christian church and cemetery (4th–7th century) to the west of the pagan cemetery (Fig. 1). The Roman walled funerary enclosure (Z4) contained a mixture of inhumation and cremation burials, probably of the 1st–4th centuries very similar to those described by Carton in the 1890s. Burials were usually accompanied with grave goods, including ceramic vessels, lamps and metalwork characteristic of the 1st–4th centuries, however, this needs to be verified though further study. In 2017–19, three seasons of documentation and limited excavation of the late antique church and cemetery were conducted under the joint direction of Moheddine Chaouali and Corisande Fenwick.

The church is a typical three-nave single-apsed basilica on an ENE–WSE orientation, which in later phases was extended by the addition of funerary annexes (Chaouali et al., 2018 for an overview; Fig. 6). The church and its annexes contained exclusively inhumation burials in built masonry tombs which were often covered by mosaic or stone epitaphs, including those of two bishops and one priest (Chaouali, 2020), and it seems that these were privileged spaces for burial. A walled enclosure adjoining the west façade of the church contains at least 98 visible tombs, including some covered by mosaics. The majority are stepped masonry tombs, which typically cover either an unmortared stone-slab burial cist or a simple undecorated sarcophagus, though they vary in quality of construction. Further west was a more elaborate late antique funerary monument: a below-ground mausoleum (perhaps a converted cistern) containing four graves dated to the second half of the 6th century. A further 47 masonry tombs and earthen graves containing inhumations have been identified around the church and enclosure. In places, the tombs are superimposed to at least three levels. Burials are universally laid out in the supine position, typically on an approximate W–E orientation following Christian burial norms, and the majority of the tombs contain a single individual. The tombs contain both males and females, ranging from young children to mature adults. Finds within the graves are extremely rare, in contrast to the burials in the Roman funerary enclosure (Z4). Ceramics, coins, glass and lead objects found around the graves suggest that the church and cemetery were in use between the 4th–7th centuries, and probably later (Chaouali et al., 2018).

### 2.2 Materials

The preservation of the remains in Bulla Regia is highly variable with some skeletons exhibiting very good preservation and others being represented by few highly weathered elements. Twenty-seven individuals were sampled for oxygen and strontium isotope analysis. Of these, fourteen late antique individuals and five Roman individuals were excavated in 2010 by the INP and eight individuals in 2017–2019 by the Bulla Regia Project. Among the late antique individuals, six are from the church and its chapels, two from the walled cemetery enclosure, four from the Mausoleum, and ten from different zones of the outer cemetery, representing a diverse sample of the entire assemblage. For comparative purposes, five individuals were sampled from the Roman funerary enclosure, two of which cannot be assigned to a specific tomb as the labels were lost post-excavation; these two individuals have not been included in the project osteoarchaeological database and thus they have not received an associated skeleton number.

For each skeleton, dental enamel was the preferred tissue for the analysis as it does not remodel and is minimally affected by diagenesis (Pellegrini et al., 2016, Price et al., 2002). Where possible, an effort was made to avoid teeth that were forming during the potential period of breastfeeding to avoid associated enrichment in ^18^O (Britton et al., 2015). Written and archaeological sources for Roman and late antique weaning habits indicate that weaning was complete by around 2.5–3 years of age (Fulminante, 2015, Keenleyside et al., 2009). Second molars were preferentially sampled, though first and third molars had to be used in cases where second molars were not preserved. For two skeletons where no molars were present, premolars were sampled instead. Only permanent teeth were used in the analysis, with one exception where a deciduous molar was analysed to maximise the sample size. Table 1 lists the sampled material, and associated sex and age-at-death of the deceased. Table 2 indicates the tooth crown (enamel) formation times for the sampled teeth.

**Table 1:** Human skeletal remains from Bulla Regia sampled for isotopic analysis (* denotes burials excavated in the 2017–19 excavations).

**Skeleton No**	**Sample No**	**Sector**	**Tomb and Human Remains Unit no** (*2010 original label where relevant)*	**Sex**	**Age-at-death**	**Tooth**
3	BR130T	Walled cemetery	T177 *(Z2 T2)*	F	MA	M2
14	BR152T	Walled cemetery	T182 *(Z2 T6)*	M?	YA	M3
6	BR54T	Outer cemetery	T47 *(Z2 T1 abords nord Sk. A)*	M	MA	M2
8	BR57T	Outer cemetery	T140 *(Z2 T5 abords nord)*	M?	MA?	M3
21	BR55T	Outer cemetery	T33 *(Z2 T1 abords sud)*	M	OA	M2
32	BR138T	Outer cemetery	T35 *(Z3, abords sud, T3 l'ouest de l'access)*	M?	MA	M3
57	BR209T	Outer cemetery	T18, 3067*	F?	YA	M3
71	BR197T	Outer cemetery	T21, 3064* cranium 2	F	YA	M2
76	BR199T	Outer cemetery	T21, 3090*	F?	MA?	M2
81	BR67T	Outer cemetery	T8, 3027*	M?	YA	M2
85	BR58T	Outer cemetery	T29, 2012*	?	infant	m2
94	BR110T	Outer cemetery	T27, 3034*	M	YA?	M3
82	BR96T	Church	T28, 1216*	M	OA	M2
77	BR231T	Church	T160, 1186*	M?	YA?	P3
24	BR48T	Chapel (Room 1)	T233 *(Z3 S4 T3 US4)*	?	YA	M2
25	BR143T	Chapel (Room 1)	T235 *(Z3 S4 T7)*	M	MA	M2
22	BR47T	Chapel (Room 12)	T236 *(Z3 S1 T1)*	M	A	M3
38	BR116T	Chapel (Room 12)	T237 *(Z3 S1 T6)*	?	2–4 yrs	M1
16	BR42T	Enclosure	*Z4 T32*	?	YA	M2
17	BR147T	Enclosure	*Z4 T23 SkA*	M?	MA	M2
18	BR46T	Enclosure	*Z4 T57*	F	YA?	*P*4
NA	BR183T	Enclosure	*UNLABELED 4*	?	?	M3
NA	BR190T	Enclosure	*UNLABELED 8 SKEL A*	?	?	M3
26	BR49T	Mausoleum	T240 *(Z1 T3)*	M?	YA	M2
27	BR51T	Mausoleum	T241 *(Z1 T4 US8)*	F	YA	M2
28	BR39T	Mausoleum	T239 *(Z1 T2 US9)*	?	11–16 yrs	M2
29	BR53T	Mausoleum	T238 *(Z1 T1 US4)*	?	10–14 yrs	M2

**Table 2:** Approximate tooth crown formation times in years. Start and end times for formation of permanent dentition are ± 6 months (Beaumont and Montgomery, 2015, following AlQahtani et al., 2010). **Denotes teeth likely to be majorly affected by a breastfeeding effect.

**Tooth**	**Approximate crown formation (years)**
m2	In utero – 0.9**
M1	0.3 – 3.5**
P3/P4	2.5 – 6.5
M2	2.5 – 8.5
M3	8.5 – 14.5

Enamel sampling took place at BioArCh at the University of York. Prior to sampling, the surface of the human teeth was first cleaned with Milli-Q water and ultrasonicated. Subsequently, the outer enamel surface was abraded with a diamond tipped handheld drill. Once the outer surface was removed, approximately 20 mg of enamel powder were collected per tooth into an Eppendorf tube using a separate drill tip for Sr analysis. Enamel was collected from across the whole surface of the tooth from just above the cementoenamel junction to the top of the occlusal surface. A further aliquot of enamel was removed from the crown using a diamond tipped rotary saw and powdered in an agate pestle and mortar for oxygen isotope analysis.

### 2.3 Isotopic analysis

**Fig. 2:**
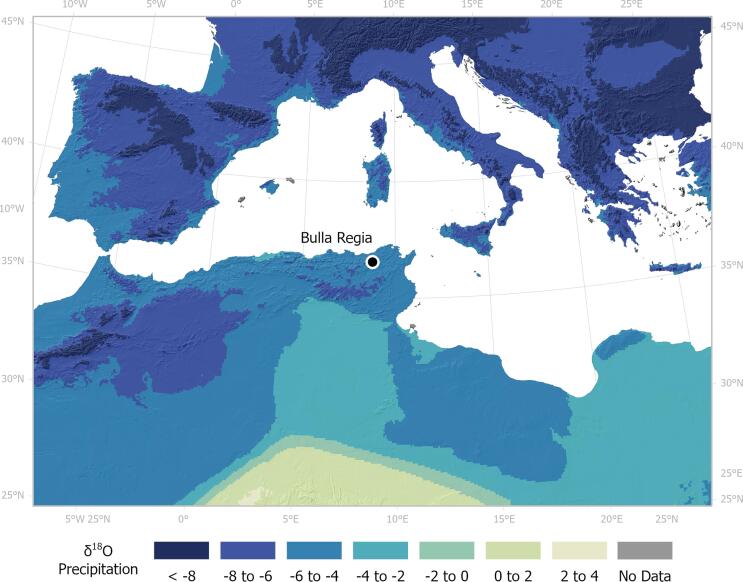
Spatial representation of δ^18^O for modern annual mean precipitation in the region overlaid onto an elevation map (GTOPO30) with the location of Bulla Regia indicated. Map created by Helen Goodchild (University of York) using ArcGIS Pro 2.8. Precipitation data derives from Bowen, 2022, Bowen and Revenaugh, 2003 using data sourced from IAEA/WMO (2022).

Isotopic analyses for the purposes of exploring migration have mostly focused on two elements: strontium and oxygen. Strontium isotope ratios vary across the world depending on the type of underlying bedrock as well as environmental contributions (e.g. sand-blown dust, sea spray, rainwater) (Bentley, 2006). Strontium from the bedrock is transferred to the soil via weathering, where it enters the food chain and is incorporated in human tissues through the consumption of plants, animals and drinking water. The ^87^Sr/^86^Sr measured in human enamel, which forms during an individual’s early life, expresses the environment where individuals spent their childhood, when tooth formation took place. Therefore, by comparing the enamel ^87^Sr/^86^Sr value against that of the burial environment, we can assess whether these individuals spent their childhood in that location or moved there at some point later in their lifetime (e.g. Willmes et al., 2018). Similarly, oxygen isotope (δ^18^O) values measured in human enamel depend primarily on drinking water and, secondarily, on ingested food. The δ^18^O values of precipitation and environmental waters vary geographically and are primarily dependent on temperature but also altitude and distance to the coast, among other factors (for a recent review, see Pederzani and Britton, 2019). Thus, by comparing the δ^18^O values of enamel against those of local waters in the region where the individuals have been found, one can predict whether these individuals are non-local (e.g. Hamre and Daux, 2016). These local waters may represent a range of water bodies including surface and underground sources which may possess variable δ^18^O values, although studies have indicated that the δ^18^O values of mammalian tissues tend to be broadly consistent with local precipitation (Pederzani and Britton, 2019). A spatial representation of δ^18^O values for modern annual mean precipitation in the region including the location of Bulla Regia is presented in Fig. 2. Approaches using both oxygen and strontium isotopes can serve to bolster interpretations made from a single chemical element alone (Britton et al., 2021; recent case studies demonstrating the successful application of combined isotopes: Depaermentier et al., 2020, Francisci et al., 2020, Ortega et al., 2021).

#### 2.3.1 Establishing a strontium isotopic baseline for northern Tunisia

As noted above, ^87^Sr/^86^Sr values in dental enamel reflect the strontium signatures of the region where an individual lived in early life, during tooth formation. Thus, enamel ^87^Sr/^86^Sr values may be compared with established baselines of bioavailable strontium in the study regions to assess whether the individuals spent their childhood in the same area where they were buried. Baselines of bioavailable strontium are mostly obtained through the measurement of modern plants, but soil, water, snails and animal teeth from taxa with small home ranges may also be used (Hartman and Richards, 2014, Lengfelder et al., 2019).

Most of northern Tunisia is characterized by sedimentary rocks starting from the Permian, while Jurassic deposits can be found in various locations. Lower and Middle Jurassic deposits include calcareous and marly sediments, while Upper Jurassic ones include deep-sea facies with radiolarites. Cretaceous deposits are also found in north Tunisia, represented by clays with ammonites and calpionellids, intercalated by calcareous and sandy deposits. Finally, reference should be made to the Numidian nappe, which crops out in northern Tunisia and forms part of the Tellian domain of the Maghreb. In our study region surrounding the archaeological site of Bulla Regia, the geology is characterized mostly by marine sediments dating from the Upper Cretaceous to the Quaternary, as well as by Triassic-Permo/Triassic argillaceous-sandy-fluviatile sediments (Schlüter, 2008).

**Fig. 3:**
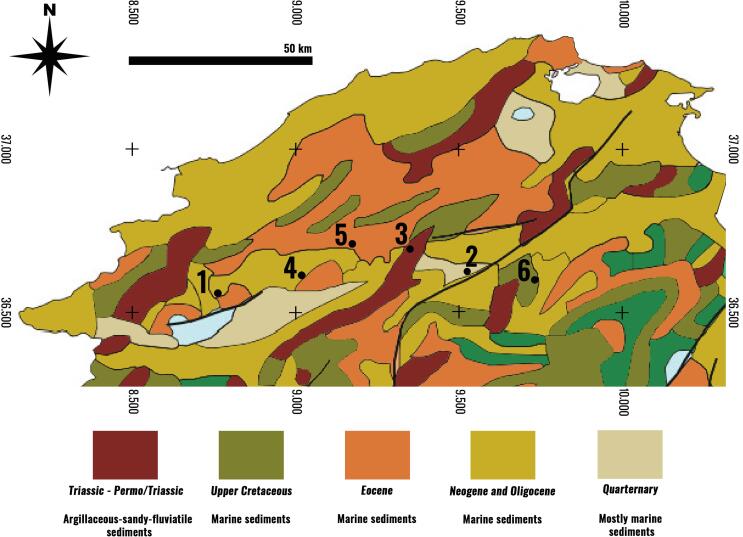
Location of sampling sites and underlying geology (geological map). Adapted from Schlüter, 2008. White zones indicate water, light blue represents Upper Jurassic limestones, clays and sandstones, while green corresponds to Lower Cretaceous sandstones, limestones, clays and sometimes terrestial intercalations. These zones have not been included in the map legend because they were not sampled.

To establish the baseline, we sampled plants with different root depth and empty snail shells from the archaeological site of Bulla Regia as well as from five other locations representing the major geological zones in northern Tunisia (Fig. 3). These locations were along a line east to the site of Bulla Regia as we drove along the landscape of Northern Tunisia, keeping maximum distance from the Tunisian-Algerian border for health and safety reasons while trying to identify sites across different geological zones. Due to limitations in time and resources, we could not sample plants and snails from several different locations belonging to the same geological zone and instead we opted for a single site per zone. An additional challenge limiting the sampling process was that northern Tunisia is heavily farmed with very limited natural landscape surviving. For this reason, every effort was made to avoid agricultural fields, as the use of fertilizers affects the strontium isotope values. To address this issue, we primarily sampled plants from hilltops, wherever possible, to avoid water-carried fertilizer from nearby cultivated fields. In addition, to avoid car pollutants, samples were collected as far from main roads as possible. This approach has the potential limitation that we have not fully captured the bioavailable variability in ^87^Sr/^86^Sr that characterises the broader region; however, it does provide an important initial baseline for a region where no other data is available.

Plants and snails have been used systematically by other scholars for the same purpose (see for example Wong et al., 2018). Note that whereas we identified different plant taxa and, as explained above, we tried to sample plants with different root depths, we could not identify different snail taxa due to lack of expertise on the topic, thus, we instead sampled whatever snail taxon was available in each site. Since snails may differ in feeding behaviour, their taxonomic identification may affect the interpretation of the results. For this reason, but most importantly for reasons related to the biases that snail values may cause to bioavailable Sr ranges, as explained below, the interpretation of the results is based both on baselines combining snail and plant values as well as on baselines using exclusively plant data.

During fieldwork, the sampled plants and snail shells were wrapped in acid free paper and stored in paper bags (for details of each sample, see Table 3). The fieldwork took place at the end of September, in parallel with the excavation at Bulla Regia, thus plants were collected after the summer months, during the region’s dry season. As a result, it was occasionally difficult to identify the sampled plants; however, a good selection of plant materials was achieved and we were able to identify most plants to the species or genus level. The identification was conducted primarily using the Field Guide to the Wild Flowers of the Western Mediterranean (Thorogood, 2016).

**Table 3:** Sampling sites and samples analysed for establishing a baseline of bioavailable ^87^Sr/^86^Sr in northern Tunisia.

**Site No**	**Site characteristics**	**Geology**	**Latitude**	**Longitude**	**Samples**
1	Bulla Regia archaeological site	Neogene and Oligocene marine sediments	36° 33′ 35′' N	8° 45′ 45′' E	Bermuda grass (*Cynodon dactylon*)
					Rush (*Juncus* sp*.*)
					Mint (*Mentha* sp.)
					Date palm (*Phoenix dactylifera*)
					Fig (*Ficus carica*)
					Snails
2	Conifer woodland, SE of Medjez el-Bab	Quaternary mostly marine sediments	36° 37′ 09′' N	9° 31′ 13′' E	Wild asparagus (*Asparagus acutifolius*)
					Acacia (*Acacia* sp.)
					Tamarisk (*Tamarix* sp.)
					Snails
3	Hilltop, uncultivated rocky ground, East of Sidi Salem Dam	Triassic-Permo/Triassic argillaceous-sandy-fluviatile sediments	36° 41′ 41′' N	9° 21′ 02′' E	Halfah grass (*Stipa tenacissima*)
					Erica (*Erica* sp.)
					Durum wheat (*Triticum durum*)
					Olive leaves (*Olea europea*)
					Snails
4	Uncultivated area among fields at East of Bou Salem, nearby small river, but uphill	Neogene and Oligocene marine sediments	36° 36′ 52′' N	9° 01′ 08′' E	Mint, wild (*Mentha* sp.)
					Olive (*Olea europea*)
					Tamarisk (*Tamarix* sp.)
					Oleander *(Nerium oleander*)
					Rush (*Juncus* sp.)
					Snails
5	Hilltop south of Beja	Eocene marine sediments	36° 42′ 38′' N	9° 10′ 26′' E	Squirting cucumber (*Ecballium elaterium*)
					Fig (*Ficus carica*)
					Unknown Grass (Poaceae)
					Olive (*Olea europea*)
					Lamiaceae
					Snails
6	Undisturbed location south-west of El Griaat	Upper Cretaceous marine sediments	36° 36′ 01′' N	9° 43′ 55′' E	Olive (*Olea europea*)
					Acacia (*Acacia* sp.)
					Lamiaceae
					Vervain (*Verbena* sp.)
					Snails

A practical limitation we faced when establishing the baseline of bioavailable ^87^Sr/^86^Sr for Tunisia was that we could not export plants for isotopic measurements since any live plants, plant parts, fruits or seeds must be accompanied by a phytosanitary certificate for the main plant pests and diseases. To obtain such a certificate from the relevant Tunisian authorities in time, we would need to know several weeks in advance exactly which plants we would be sampling; however, in our case this was impossible to determine until a few days before our departure from the country. To overcome this issue, and since ashing plants is the first step in the preparation of organic samples for isotopic analysis, we decided to expose our plant samples to an open wood fire in the field, so that a mixture of charcoal and ash could be exported without any special license.

To test whether charring/ashing plants in an open fire would result in any substantial alteration in their ^87^Sr/^86^Sr values compared to a controlled laboratory furnace, we conducted an experimental study in South Africa prior to fieldwork. We collected six plant species from Cape Town, South Africa with different root depths: grass (*Cynoclon dactylon*), palmiet (*Prionum serratum*), palm (*Phoenix reclinata*), forest fig (*Ficus craterostoma*), fig (*Ficus sycomorus*), and sedge (*Cyperus* sp.). Each sample was divided into two parts; one part was first exposed to an open fire to produce a mixture of charcoal and ashes, which is what we expected to obtain from an open fire once in the field in Tunisia, while the other was ashed in a laboratory furnace. For the open fire charring/ashing, plants were placed inside aluminium foil, tightly closed to avoid contamination. Subsequently, the aluminium foil ‘parcels’ were placed in proximity to an open wood fire, and closely monitored and removed when converted to charcoal and ash, eliminating any potentially hazardous biological material. Once transported to the lab, these samples were fully ashed in covered porcelain crucibles at 650 °C, following standard procedures (e.g. Wong et al., 2018). The samples that were only ashed in a laboratory setting were first dried at 60 °C and then ashed in covered porcelain crucibles at 650 °C, following standard procedures (e.g. Wong et al., 2018). The end result was two duplicate sets of ashed plant samples, one solely processed in a controlled laboratory setting and the other having undergone an initial heat exposure step in an open fire followed by further ashing in a controlled laboratory setting. The results of this experiment supported the lack of any contamination, as detailed below.

Once in the field in Tunisia, the same procedures adopted in South Africa were followed to process on site the plants collected (see Table 3). The plants were first gently cleaned with pure water, to remove soil contaminants and dust. Then they were gently broken into smaller parts and placed inside a parcel made of several layers of aluminium foil. A fire was set up in Bulla Regia using palm wood. The fire was located close to a fire extinguisher, and inside a large shallow pit, where it could be controlled. Once fire embers were obtained, one parcel at a time was placed on an aluminium foil tray near the embers to avoid direct contact with the flames. The fire was maintained throughout the process to continue generating heat and embers. The parcels were constantly monitored and their outer foil inspected to ensure no holes formed causing loss of material or the contamination of their content. Smoke was observed in all the parcels initially, but then the smoke production stopped and the plant content was reduced to small pieces of charcoal and ashes within 2 to 5 h. Once the process was completed, up to 100 mg of charcoal/ash per plant were transferred in labelled plastic tubes. Thus, this process reduced not only the risk of sending plant matter abroad, but also the volume of the material as in our study, for example, less than 5 g of charcoal/ash were exported in total.

#### 2.3.2 Strontium isotopic preparation and measurements

The chemical preparation of all samples (enamel, plant charcoal/ash, snail shells) and the ^87^Sr/^86^Sr measurements took place at the Department of Geological Sciences, University of Cape Town, and followed the procedure and referencing values (SRM987 ^87^Sr/^86^Sr of 0.710255) described in Leggett et al. (2021). Note that, once at the lab, the plant charcoal/ash from Tunisia was placed in covered porcelain crucibles inside furnaces at 650 °C to ensure the full combustion of any potential remnant organics. Mann-Whitney *U* tests were used for comparisons between males and females, and between plant and snail values per site. The significance level was set at α = 0.05. To identify non-locals, the obtained ^87^Sr/^86^Sr values were compared against the bioavailable strontium isotope baseline of Bulla Regia and surrounding regions from northern Tunisia produced using modern plants and snails, as described above.

The repeated measurement of reference materials as unknowns along with the samples from this study yielded good results. Reference material BHVO-2 was processed along with the ashed plant samples and the average ^87^Sr/^86^Sr value of 0.703491 ± 0.000012 (n = 4) agreed with accepted values (0.703484 ± 0.000039, long-term UCT average n = 298; 0.703478 ± 0.000034, GeoReM, Jochum et al., 2005). In-house carbonate reference material NM95 was processed with the snail and enamel samples, with the respective ^87^Sr/^86^Sr values of 0.708899 ± 0.000032 (n = 3) and 0.708911 ± 0.000039 (n = 4) agreeing with long-term results for this reference material in this facility (0.708911 ± 0.000040, long-term UCT average, n = 414). All total procedural Sr blanks were < 250 pg and therefore negligible.

#### 2.3.3 Oxygen isotopic preparation and measurements

Enamel carbonate pre-treatment took place at BioArCh at the University of York following the method of Miller et al. (2018). Powdered dental enamel was pre-treated by adding 0.1 ml of 0.1 M of acetic acid per mg of enamel to each sample for 10 min. The samples were then rinsed three times with ddH_2_O before being freeze-dried. Enamel apatite δ^18^O was analysed at IsoAnalytical, Cheshire, UK, by Continuous Flow-Isotope Ratio Mass Spectrometry (CFIRMS) using a Europa Scientific 20–20 mass spectrometer. Powdered samples and controlled carbonates were weighed into ExetainerTM tubes (Labco, UK) which were then flushed with 99.995 % helium, and the samples converted to carbon dioxide by injecting phosphoric acid. Control material analysed alongside the samples included IA-R022 (IsoAnalytical working standard calcium carbonate, δ^18^O_V-PDB_ = –22.69 ‰, n = 10), NBS-18 (carbonatite, δ^18^O_V-PDB_ = –23.20 ‰, n = 4) and IA-R066 (chalk, δ^18^O_V-PDB_ = –1.52 ‰, n = 4). Standard deviations of repeated measurements of these standards were ≤ 0.11 ‰ for δ^18^O. Carbonate δ^18^O_V-PDB_ values are reported as δ^18^O_VSMOW_ after Coplen (1988). To identify potential migrants, δ^18^O_VSMOW_ values were converted to drinking water (dw) using the equation of Chenery et al. (2012) to compare to modern local water values from the GNIP database (IAEA/WMO, 2022). The complications of equation selection and error propagation when predicting drinking water values however, need to be acknowledged (see Lightfoot and O'Connell, 2016 and Pederzani and Britton, 2019 for discussion). It should also be borne in mind that the comparison with modern water values cannot take into account any potential changes with climate and therefore δ^18^O_precipitation_ though time.

## 3 Results

### 3.1 Experimental testing of the effect of open fire plant charring/ashing

**Fig. 4:**
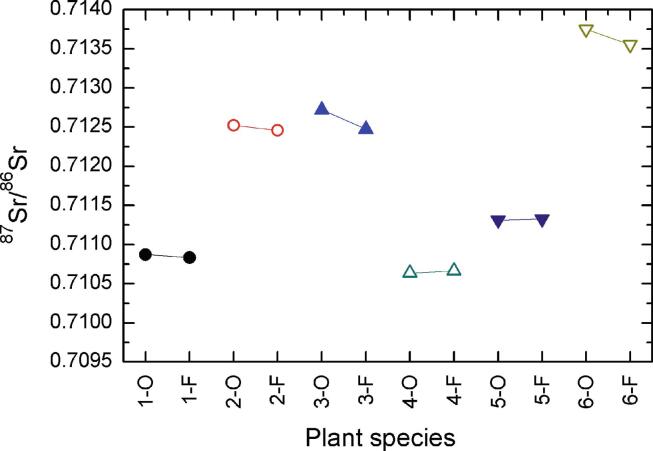
Visualization of ^87^Sr/^86^Sr values for differentially treated plants; notice the clear pairs formed by the values of the same plants Key: O: open fire & furnace; F: furnace; 1 = Grass (*Cynoclon dactylon*); 2 = Palmiet (*Prionum serratum*); 3 = Palm (*Phoenix reclinata*); 4 = Forest Fig (*Ficus craterostoma*); 5 = Fig (*Ficus sycomorus*); 6 = Sedge (*Cyperus* sp.).

The ^87^Sr/^86^Sr values measured on plants that had initially been exposed to an open fire prior to being ashed in the lab were almost identical to those of the same plants ashed in a laboratory furnace only, following the standard procedure of samples preparation for strontium isotopic analysis (Table 4). When plotted, it is seen that despite minimal differences, the values from the same plants (charred/ashed in open fire vs ashed in furnace) form distinct pairs that separate each type from the others (Fig. 4). A Wilcoxon signed ranks test further supported the lack of a statistically significant difference between the paired values of open fire charred/ashed vs furnace ashed plants (*Z*: −1.572, p = 0.156).

**Table 4:** Results of differential treatment of plant samples prior to Sr isotopic measurement.

**Plant sample**	**Open fire & Furnace**		**Furnace**	
	**^87^Sr/^86^Sr**	**±2s**	**^87^Sr/^86^Sr**	**±2s**
Grass (*Cynoclon dactylon*)	0.710871	0.000012	0.710834	0.000011
Palmiet (*Prionum serratum*)	0.712522	0.000010	0.712459	0.000012
Palm (*Phoenix reclinata*)	0.712720	0.000013	0.712474	0.000012
Forest Fig (*Ficus craterostoma*)	0.710636	0.000013	0.710665	0.000011
Fig (*Ficus sycomorus*)	0.711312	0.000010	0.711328	0.000013
Sedge (*Cyperus* sp.)	0.713750	0.000009	0.713553	0.000011

### 3.2 Bioavailable strontium isotopic baseline for northern Tunisia

The ^87^Sr/^86^Sr values in Bulla Regia, which lies on Neogene and Oligocene marine sediments, range from 0.708075 to 0.708441 (Table 5). In sampling site 2, consisting of Quaternary mostly marine sediments, the values are overall lower, spanning 0.707973 to 0.708261, while in sampling site 3, characterized by Triassic-Permo/Triassic argillaceous-sandy-fluviatile sediments, they are higher, ranging from 0.708281 to 0.710309. In sampling site 4, which is in the same geological zone as Bulla Regia, the ^87^Sr/^86^Sr values range from 0.708291 to 0.708552, in sampling site 5, characterized by Eocene marine sediments, from 0.708111 to 0.708729, and, finally, in sampling site 6, lying on Upper Cretaceous marine sediments, from 0.708394 to 0.708734. All geological zones consisting of marine sediments exhibit largely comparable values, with older deposits showing generally higher values, as expected.

**Table 5:** Bioavailable ^87^Sr/^86^Sr values in Bulla Regia and other northern Tunisian locations across different geological zones.

**Site No**	**Samples**	**^87^Sr/^86^Sr**	**±2s**
1	Bermuda grass (*Cynodon dactylon*) 1	0.708420	0.000010
	Bermuda grass (*Cynodon dactylon*) 2	0.708405	0.000014
	Rush (*Juncus* sp.) 1	0.708263	0.000010
	Rush (*Juncus* sp.) 2	0.708269	0.000014
	Mint, wild (*Mentha* sp*.*) 1	0.708271	0.000016
	Mint, wild (*Mentha* sp*.*) *2*	0.708334	0.000015
	Date palm (*Phoenix dactylifera*) 1	0.708124	0.000010
	Date palm (*Phoenix dactylifera*) 2	0.708441	0.000013
	Fig (*Ficus carica*) 1	0.708183	0.000014
	Fig (*Ficus carica*) 2	0.708191	0.000009
	Snail 1	0.708092	0.000013
	Snail 2	0.708114	0.000010
	Snail 3	0.708075	0.000010
	Snail 4	0.708088	0.000012
	Snail 5	0.708085	0.000011
2	Wild asparagus (*Asparagus acutifolius*) 1	0.708191	0.000020
	Wild asparagus (*Asparagus acutifolius*) 2	0.708239	0.000012
	Acacia (*Acacia* sp.)	0.708261	0.000010
	Tamarisk (*Tamarix* sp.)	0.708208	0.000013
	Snail 1	0.708009	0.000011
	Snail 2	0.707989	0.000009
	Snail 3	0.707998	0.000014
	Snail 4	0.707987	0.000014
	Snail 5	0.707973	0.000014
3	Halfah grass (*Stipa tenacissima*) 1	0.709448	0.000014
	Halfah grass (*Stipa tenacissima*) 2	0.710309	0.000011
	Erica (*Erica* sp.) 1	0.709484	0.000011
	Erica (*Erica* sp.) 2	0.709296	0.000014
	Durum wheat (*Triticum durum*)	0.709312	0.000010
	Olive (*Olea europea*)	0.709011	0.000012
	Snail 1	0.708381	0.000011
	Snail 2	0.708281	0.000012
	Snail 3	0.708422	0.000009
	Snail 4	0.708472	0.000012
4	Mint, wild (*Mentha* sp.)	0.708455	0.000010
	Olive (*Olea europea*)	0.708552	0.000011
	Tamarisk (*Tamarix* sp.)	0.708356	0.000011
	Oleander (*Nerium oleander*)	0.708291	0.000012
	Rush (*Juncus* sp.)	0.708401	0.000011
	Snail 1	0.708466	0.000010
	Snail 2	0.708418	0.000014
	Snail 3	0.708494	0.000011
	Snail 4	0.708420	0.000008
	Snail 5	0.708458	0.000011
5	Squirting cucumber (*Ecballium elaterium*)	0.708512	0.000010
	Fig (*Ficus carica*)	0.708356	0.000010
	Unknown grass (*Poaceae*)	0.708729	0.000011
	Olive (*Olea europea*)	0.708401	0.000010
	Vervain (*Lamiaceae*)	0.708528	0.000011
	Snail 1	0.708111	0.000011
	Snail 2	0.708137	0.000010
	Snail 3	0.708212	0.000014
	Snail 4	0.708229	0.000011
	Snail 5	0.708185	0.000014
6	Olive (*Olea europea*)	0.708476	0.000012
	Acacia (*Acacia* sp.)	0.708496	0.000013
	Vervain (*Lamiaceae*)	0.708734	0.000012
	Vevain (*Verbena* sp.)	0.708530	0.000013
	Snail 1	0.708420	0.000012
	Snail 2	0.708394	0.000011
	Snail 3	0.708437	0.000015
	Snail 4	0.708424	0.000010
	Snail 5	0.708481	0.000011

Note that in all sampling sites, expect for sampling site 4, snails exhibit significantly lower ^87^Sr/^86^Sr values than plants (Table 6). This finding agrees with several previous studies that identified lower radiogenic ^87^Sr/^86^Sr ratios in snail shells compared to other sources of bioavailable Sr, and have interpreted it by the fact that ^87^Sr/^86^Sr values of land snails are commonly shifted towards soil carbonates or rainwater values, rather than bulk local soil values (Britton et al., 2020, Lugli et al., 2022, Maurer et al., 2012, Yanes et al., 2008).

**Table 6:** Comparison between snail and plant ^87^Sr/^86^Sr values.

**Site No**	**Average snail**	**Average plant**	**Mann-Whitney *U***	**z**	**p-value**
1	0.708091	0.708290	0.000	−3.001	0.001
2	0.707991	0.708225	0.000	−2.327	0.016
3	0.708389	0.709477	0.000	−2.452	0.010
4	0.708451	0.708411	7.000	−1.045	0.310
5	0.708175	0.708505	0.000	−2.507	0.008
6	0.708431	0.708559	1.000	−2.082	0.032

### 3.3 Identifying non-locals in Bulla Regia

The ^87^Sr/^86^Sr values for the Bulla Regia individuals are presented in Table 7. These values range between 0.707971 and 0.708986 with a mean value of 0.708393 ± 0.000196 (n = 27). Males and females exhibit comparable values with those for males being slightly higher (average ^87^Sr/^86^Sr value of 0.708441 ± 0.00018 for males (n = 13) and 0.708310 ± 0.000179 for females (n = 6)). A Mann-Whitney test did not find a statistically significant difference between males and females (Mann Whitney *U*: 25, z: −1.184, p = 0.2364). In addition, an analysis of the coefficient of variation suggested equal variation in male and female strontium signatures (males: 0.026 %, females: 0.025 %).

**Table 7:** ^87^Sr/^86^Sr, δ^18^O_VSMOW_(carb) and δ^18^O_VSMOW_(dw) values for the individuals from Bulla Regia. δ^18^O_carb_ are reported as VSMOW in the text.

**Skeleton No**	**Phase**	**Sex**	**^87^Sr/^86^Sr**	**±2s**	**δ^18^O (‰)** **VPDB**	**δ^18^O (‰)** VSMOW*	**δ^18^O_dw_ (‰)** VSMOW** (estimated)
3	Late antique	F	0.708509	0.000014	−3.1	27.7	−4.5
14	Late antique	M?	0.708361	0.000009	−3.2	27.6	−4.7
6	Late antique	M	0.708278	0.000009	−3.4	27.4	−5.0
8	Late antique	M?	0.708578	0.000011	−3.2	27.6	−4.7
21	Late antique	M	0.708419	0.000010	−3.4	27.4	−5.1
32	Late antique	M?	0.708296	0.000010	−2.7	28.1	−4.0
57	Late antique	F?	0.708347	0.000012	−3.1	27.7	−4.6
71	Late antique	F	0.708315	0.000010	−3.4	27.4	−5.0
76	Late antique	F?	0.708371	0.000010	−3.4	27.4	−5.1
81	Late antique	M?	0.708381	0.000012	−4.3	26.5	−6.5
85	Late antique	?	0.708088	0.000011	−1.9	28.9	−2.7
94	Late antique	M	0.708339	0.000009	−3.6	27.2	−5.4
82	Late antique	M	0.708387	0.000010	−3.0	27.8	−4.4
77	Late antique	M?	0.708403	0.000012	−2.8	28.0	−4.1
24	Late antique	?	0.708851	0.000011	−2.7	28.2	−3.8
25	Late antique	M	0.708986	0.000010	−1.9	28.9	−2.7
22	Late antique	M	0.708341	0.000011	−3.1	27.7	−4.6
38	Late antique	?	0.708441	0.000023	−1.6	29.2	−2.1
16	Roman	?	0.708357	0.000012	−4.1	26.7	−6.2
17	Roman	M?	0.708417	0.000012	−2.0	28.8	−2.8
18	Roman	F	0.708348	0.000013	−3.6	27.2	−5.5
NA	Roman	?	0.708242	0.000014	−3.0	27.8	−4.4
NA	Roman	?	0.708230	0.000012	−2.9	28.0	−4.2
26	Late antique	M?	0.708546	0.000010	−3.1	27.7	−4.6
27	Late antique	F	0.707971	0.000009	−1.8	29.0	−2.5
28	Late antique	?	0.708418	0.000012	−3.8	27.0	−5.7
29	Late antique	?	0.708404	0.000014	−2.7	28.2	−3.8

*VPDB converted to VSMOW following Coplen (1988).

**Drinking water values converted using equations in Chenery et al. (2012).

The ^87^Sr/^86^Sr values in Bulla Regia for plants and snails range from 0.708075 to 0.708441 (Table 5). Six individuals exhibit values beyond this range (3, 8, 24, 25, 26, 27) while an additional individual (38) has a value on the upper limit of the bioavailable baseline (Table 7 and Fig. 4). The non-local individuals represent both males and females, young and middle adults, as well as a young child. In addition, these individuals originate in different sections of the church and cemetery, supporting the lack of a spatial segregation of locals and non-locals in mortuary treatment.

Though the evidence suggests that these outliers may reflect the inward migration of non-local individuals, individuals 3, 26 and 38 could have originated from any geological zone represented by sampling sites 3 to 6; individual 27 does not match any of the sites sampled though her value is rather close to the lower limit for site 2; individual 8 could have originated from any geological zone represented by sites 3, 5 or 6; and individuals 24 and 25 could have originated from any geological zone represented by site 3. In summary, all individuals were either local to Bulla Regia or could have originated from other parts of northern Tunisia, without being able to exclude the possibility of them originating from areas with a similar ^87^Sr/^86^Sr baseline further away.

Note that when we exclude snails from the bioavailable baseline given the fact that their ^87^Sr/^86^Sr value often reflects soil carbonates or rainwater values, the only difference from the above results is that individual 85 is also non-local to Bulla Regia. In addition, with these more restricted plant baselines, individuals 27 and 85 do not match any of the geological zones sampled so they may represent migrants arriving in Bulla Regia from further away (though issues of the representativeness of the available baseline must be considered as outlined in section 2.3.1). Individual 85 was represented by a deciduous tooth. Since the Sr isotope composition of deciduous teeth reflects that of the individual’s mother (Lugli et al., 2017, Lugli et al., 2019), this value indicates that the child had a non-local mother and was non-local itself (since the crown of the second deciduous molar forms in utero and within the first year of life – Table 2). Note that the strontium isotopic value for individual 85 is distinct from that of the two non-local females identified in the assemblage (individuals 3 and 27), thus it is unlikely that one of these is the mother of individual 85. With the new baselines excluding snails, individuals 24 and 25 also do not match any specific zone sampled, but their values lie between those of geological zone 3 and all others.

The δ^18^O_VSMOW_(carb) values for the Bulla Regia population range from 26.5 ‰ to 29.2 ‰ (range = 2.7 ‰), with a mean of 27.8 ± 0.7 ‰ (1 s), see Table 7. Two individuals under the age of four years old (individuals 38 and 85) possess some of the highest δ^18^O_VSMOW_(carb) values that likely indicate a breastfeeding effect (Britton et al., 2015) and will therefore not be included in interpretations surrounding mobility. Removing these individuals leads to a slight narrowing of the overall range (2.5 ‰) and distribution of values (mean of 27.7 ± 0.6 ‰, 1 s). Males (n = 13) and females (n = 6) possess similar distributions of δ^18^O_VSMOW_(carb) values (male mean 27.8 ± 0.6 ‰, 1 s = female mean = 27.7 ± 0.7 ‰, 1 s), with no statistical difference between them (Mann–Whitney *U*: 33.5, z: 0.43872, p = 0.66).

**Fig. 5:**
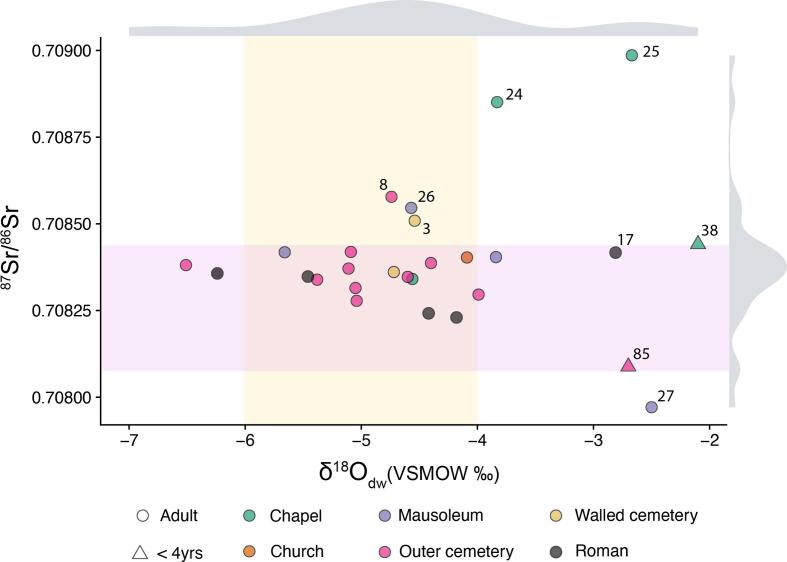
Scatterplot with marginal density distributions plotted for ^87^Sr/^86^Sr and δ^18^O_VSMOW_(dw) values converted to drinking water for Bulla Regia individuals. Purple shading indicates the local ^87^Sr/^86^Sr baseline based on modern plants and snails. Yellow shading represents the estimated local δ^18^O_VSMOW_(dw) calculated from modern precipitation values using Bowen (2022), Fig. 1.

The predicted isotopic values of drinking water, δ^18^O_VSMOW_(dw), for individuals over 4yrs old range from −6.5 ‰ to −2.5 ‰ (Table 7). Most of these individuals fit within the estimated range of δ^18^O for mean annual modern precipitation in the local region (∼-6 to −4 ‰, Fig. 4 and Fig. 5). Two individuals possess δ^18^O_VSMOW_(dw) values that fall just below the local range (<-6‰) that are broadly consistent with areas of high elevation to the south and west of the site (Fig. 5) and possess ^87^Sr/^86^Sr values in keeping with the local biosphere. Five have more elevated δ^18^O_VSMOW_(dw) values than the estimated local range (excluding individuals 38 and 85), of which three are also outside the predicted local range for Sr (individuals 24, 25 and 27). Individual 27 with a δ^18^O_VSMOW_(dw) value of −2.5 ‰ is the only statistical outlier calculated using Tukey’s 1.5IQR method (Lightfoot and O’Connell, 2016). One Roman individual (17) possesses a Sr value congruent with the local biosphere but a δ^18^O_VSMOW_(dw) value elevated above the local range (−2.8 ‰). These more elevated δ^18^O_VSMOW_(dw) values are suggestive of an origin from a warmer climate found further south or east of Bulla Regia, including areas of modern-day southern Tunisia, Algeria, Libya and Egypt (Fig. 5) or indeed the Middle East. However, given the potential for higher δ^18^O values to derive from human alteration of ingested water (e.g. brewing, boiling, vinification, Brettell et al., 2012), it is those individuals who possess values for both ^87^Sr/^86^Sr and δ^18^O_VSMOW_(dw) outside the estimated local range that are the most convincing potential non-locals among the oxygen isotope dataset.

**Fig. 6:**
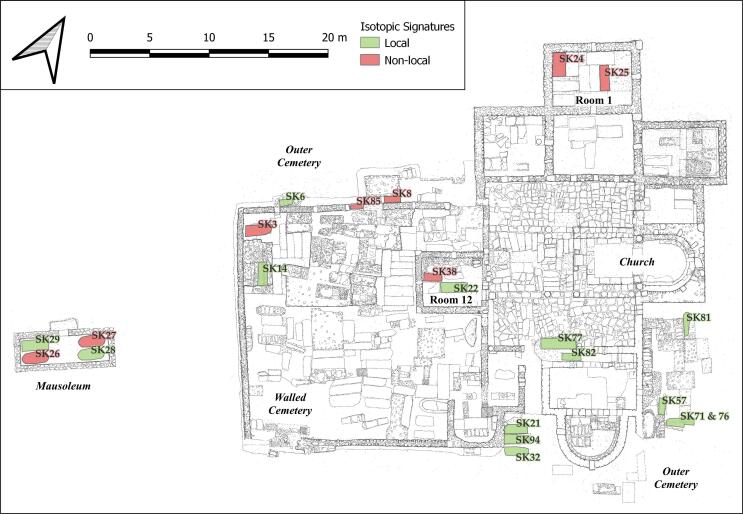
Plan of the church and cemetery showing the location of sampled skeletons: non-local signatures in red and local signatures in green (Bulla Regia Archaeological Project).

## 4 Discussion

Our results suggest that at Bulla Regia, at least seven out of the 27 individuals examined (26 %) appear not to have originated in this town, possessing ^87^Sr/^86^Sr values outside the local bioavailable range that are compatible with other parts of north Tunisia. When adopting a more conservative bioavailable ^87^Sr/^86^Sr baseline using only plants and excluding snails, the number of nonlocals is increased to eight and two of the nonlocal individuals (27 and 85) exhibit values too low to match any of the sampled zones in northern Tunisia. Nonetheless, given the above described limitations in our sampling strategy, we cannot exclude the possibility that these two individuals match some part of the region that has not been sampled. The δ^18^O_VSMOW_(dw) values for three of these individuals (8, 26, 3) are in keeping with the modern local range for precipitation and are also congruent with a potential origin in north Tunisia. Three other potential non-locals (24, 25, 27) however, possess elevated δ^18^O_VSMOW_(dw) values that could potentially indicate an origin from a warmer climate and this allows us to infer a potential origin from an area further south and east. Two additional individuals (16, 81) possess ‘local’ ^87^Sr/^86^Sr signatures but slightly lower δ^18^O_VSMOW_(dw) values than the estimated local range which may be indicative of an origin from a cooler climate with similar geology. Given the potential for environmental and subsurface waters to deviate from local precipitation values (Pederzani and Britton, 2019), however, it cannot be ruled out that these individuals may be locals that relied upon a water source with lower δ^18^O_VSMOW_(dw) values such as water originating from high elevations in the area (Fig. 2).

Given these results, mobility need not have been long-distant for most of the potential non-locals at Bulla Regia, though we cannot exclude the possibility that the individuals originated from a much more distant location with similar δ^18^O_VSMOW_(dw) values and bioavailable ^87^Sr/^86^Sr signatures. The first author (E.N.) recently created SrIsoMed, a searchable database of published ^87^Sr/^86^Sr values from countries that have coastlines on the Mediterranean Sea, accessible at https://srisomed.emmebioarch.com. According to this database and the values for modern δ^18^O_precipitation_ (Fig. 2), Bulla Regia non-locals have values that match the bioavailable ^87^Sr/^86^Sr and δ^18^O_precipitation_ in various locations across the western, central and eastern Mediterranean. Thus, whilst the simplest explanation is that most of the late antique individuals buried in the Christian church and cemetery were locals and most of the non-locals originated from neighbouring regions, particularly those with a warmer climate, further research is required.

Examining the spatial and chronological distribution of individuals is revealing: all five Roman individuals from the funerary enclosure have ^87^Sr/^86^Sr signatures in keeping with the local biosphere, however, the δ^18^O_VSMOW_(dw) values of two individuals (17, 16) lie either side of the estimated local range, with higher and lower δ^18^O_VSMOW_(dw) values, respectively. This suggests that while the majority of people buried in this enclosure may be local, there is some evidence for potential non-local origins. The sample size is small given the large size of the Roman cemetery and the variety of tomb types including temple mausolea and cupola tombs (Carton, 1890, Chaouali et al., 2018). It is also restricted to a single funerary enclosure characterised by simple graves and limited grave goods with no inscribed tomb markers. These preliminary results cannot, therefore, support or refute Thébert’s (1973) onomastic study of Bulla Regia’s inscriptions, which inferred a largely indigenous Roman era population.

The spatial distribution of the isotopic signatures of late antique individuals is more striking still (Fig. 6). While the majority of those who are buried in the simpler graves of the outer cemetery and the two burials (77, 82) from the earliest phase of the church have local signatures, three of four individuals sampled within the funerary chapels (privileged spaces) and two of four individuals sampled from an outlying mausoleum have non-local signatures. In Room 1, one of the later funerary chapels to be built, individuals 24 and 25 have non-local signatures for both ^87^Sr/^86^Sr and δ^18^O_VSMOW_(dw). Both were commemorated by elaborate mosaics, including in the case of individual 25, a mosaic (M25) depicting Jonah and the whale. In Room 12, a late chapel added opposite the apse, individual 38 (2–4yrs old) has non-local ^87^Sr/^86^Sr signature and is buried in the funerary chapel of bishop Procesius (individual 22) who has a local signature. In a mausoleum immediately to the west of the Christian walled cemetery dated to the sixth century, the adult individuals 26 and 27 had non-local ^87^Sr/^86^Sr, although the other two graves of young adults here have local signatures and individual 26 has a δ^18^O_VSMOW_(dw) value in keeping with the majority of the population. Strikingly, these two individuals both were buried with goods: individual 26 was buried with nine *nummi* of Justin II (r. 565–574) and individual 27 (a female) was buried with fine earrings. In the walled cemetery, individual 3 with a non-local ^87^Sr/^86^Sr signature was buried in a tomb that was originally covered by a mosaic. Individual 8 is outside the enclosure and is perhaps the exception, however, he was placed in a well-built tomb. There seems to be a pattern therefore of non-local individuals being buried in tombs of higher quality or privileged places, such as within funerary chapels or a free-standing mausoleum. We might therefore see this as a reflection of the mobility of wealthier town-dwellers in late antiquity, particularly perhaps along the Carthage-Hippo route, though further research is needed.

The shortage of comparative studies limits the placement of the current results in a broader context. Nonetheless, the results from Bulla Regia, identifying potentially at least 26 % of the assemblage as non-local as well as favouring regional mobility, reveal a pattern largely consistent with late antique mobility in other parts of the Mediterranean. Nonetheless, studies elsewhere in the Mediterranean have found very diverse results, indicating that mobility patterns vary across time and space as the result of different social and economic forces. Early Christian and Late Byzantine populations from Cyprus show movements of people around the island, but not necessarily from further afield (Nikita et al., 2021). Increased numbers of non-local individuals from the Roman to Byzantine periods in Hierapolis indicate its importance as a Christian pilgrimage site in the 9th–13th centuries (Wong et al., 2018). On the other hand, isotopic data from Corinth, Greece, show a long-term process of non-locals being assimilated into the local culture (Kennedy, 2016), as well as migrations of populations around the Italian Peninsula when economic conditions were poor (e.g. Maxwell, 2019, Paladin et al., 2020, Temkina, 2021). In one exceptional case, non-local individuals identified in 5th–7th century Wales have been hypothesized to be Byzantines from the Mediterranean (Hemer et al., 2013). At Bulla Regia, though the sample size is small, the distribution of non-local and local individuals in the church and cemetery suggests that those who were buried in privileged tombs were more likely to be mobile. These preliminary results prompt further investigation at Bulla Regia to clarify whether social status may have played a role in mobility.

## CRediT authorship contribution statement

**Efthymia Nikita:** Conceptualization, Data curation, Formal analysis, Funding acquisition, Investigation, Methodology, Project administration, Resources, Software, Supervision, Writing – original draft, Writing – review & editing. **Michelle Alexander:** Conceptualization, Data curation, Formal analysis, Investigation, Methodology, Resources, Software, Supervision, Validation, Writing – review & editing. **Samantha Cox:** Data curation, Investigation, Writing – review & editing. **Anita Radini:** Conceptualization, Investigation, Methodology, Writing – review & editing. **Petrus Le Roux:** Formal analysis, Methodology, Resources, Software, Validation, Writing – review & editing. **Moheddine Chaouali:** Conceptualization, Data curation, Funding acquisition, Investigation, Project administration, Supervision, Writing – review & editing. **Corisande Fenwick:** Conceptualization, Data curation, Formal analysis, Funding acquisition, Investigation, Methodology, Project administration, Resources, Software, Supervision, Writing – original draft, Writing – review & editing.

## Declaration of Competing Interest

The authors declare that they have no known competing financial interests or personal relationships that could have appeared to influence the work reported in this paper.
